# Tech-Savvy Beef Cattle? How Heifers Respond to Moving Virtual Fence Lines

**DOI:** 10.3390/ani7090072

**Published:** 2017-09-18

**Authors:** Dana L. M. Campbell, Jim M. Lea, William J. Farrer, Sally J. Haynes, Caroline Lee

**Affiliations:** 1 Agriculture and Food, CSIRO, New England Highway, Armidale, NSW 2350, Australia; jim.lea@csiro.au (J.M.L.); caroline.lee@csiro.au (C.L.); 2 Agersens, Pty Ltd., Melbourne, VIC 3000, Australia; will.farrer@agersens.com (W.J.F.); shaynes@agersens.com (S.J.H.)

**Keywords:** GPS, technology, welfare, associative learning, activity, behavioural patterns

## Abstract

**Simple Summary:**

Physical fences are not always possible, thus automated technology called “virtual fencing” provides a potential solution. Virtual fencing uses Global Positioning System (GPS) technology and animals wear collar devices. As animals approach the virtual fence line, the collar emits an audio tone; if the animals walk further forward, they receive an electrical stimulus. If the animal turns around after the audio tone, they receive no electrical stimulus. However, no studies to date have looked at how animals respond when virtual fences have moved to different paddock locations. Virtual boundaries were set up to restrict six beef cattle wearing collars to different paddock areas. Within a few days, the animals were able to avoid the electrical stimulus by learning to turn away from the fence when they heard the audio tone. Over several weeks, the virtual fence was moved to three different locations within the paddock, and the animals rapidly learned it had moved, turning away at the audio tone the majority of the time. This shows that animals can learn the different collar signals and avoid moving virtual boundaries via the audio tone. The application of virtual fencing to farms enables improved animal management and animal exclusion from environmentally sensitive areas.

**Abstract:**

Global Positioning System (GPS)-based virtual fences offer the potential to improve the management of grazing animals. Prototype collar devices utilising patented virtual fencing algorithms were placed on six Angus heifers in a 6.15 hectare paddock. After a “no fence” period, sequential, shifting virtual fences restricted the animals to 40%, 60%, and 80% of the paddock area widthways and 50% lengthways across 22 days. Audio cues signaled the virtual boundary, and were paired with electrical stimuli if the animals continued forward into the boundary. Within approximately 48 h, the cattle learned the 40% fence and were henceforth restricted to the subsequent inclusion zones a minimum of 96.70% (±standard error 0.01%) of the time. Over time, the animals increasingly stayed within the inclusion zones using audio cues alone, and on average, approached the new fence within 4.25 h. The animals were thus attentive to the audio cue, not the fence location. The time spent standing and lying and the number of steps were similar between inclusion zones (all *p* ≥ 0.42). More lying bouts occurred at the 80% and lengthways inclusion zones relative to “no fence” (*p* = 0.04). Further research should test different cattle groups in variable paddock settings and measure physiological welfare responses to the virtual fencing stimuli.

## 1. Introduction

The effective management of grazing animals is an ongoing challenge for producers. The constant drive towards improved animal efficiency and optimal use of available land and resources can be hindered by vast land areas and limited contact with animals. Perennial advances in technology, combined with an improved understanding of animal behaviour and welfare facilitate the reality of virtual fences as a sustainable fencing solution with potential reductions in labour and physical fencing costs [1]. Virtual fences are defined as boundaries, enclosures, or barriers without a physical barrier [2], and may rely on the animal wearing a device. The animal is typically trained through associative learning of a neutral audio stimulus with a negative or aversive stimulus, normally an electrical stimulus [2]. While the last approximately four decades have seen the development and testing of multiple types of virtual fencing systems, including both Global Positioning System (GPS)-based or physical cable-based systems, few of these researched devices are commercially available or widely used on-farm [2].

A patented, automated virtual fencing algorithm that utilises GPS technology and requires the animal to wear a collar device was developed through the Commonwealth Scientific and Industrial Research Organisation (CSIRO), Australia [3,4]. This algorithm operates by emitting an audio tone as the animal approaches the virtual fence boundary; if the animal stops or turns away in response to the audio cue, no electrical stimulus is applied, but if the animal continues forward it receives an aversive electrical stimulus. Associative learning between the audio cue and electrical stimulus has been successfully demonstrated in experimental settings using remote-controlled, manual virtual fencing collars [5].

In a recent trial conducted with the same animals as used in the current study, automated experimental prototype virtual fencing collars were able to successfully deliver appropriate cues to exclude individual animals from accessing a feed reward (hay) in a small test paddock [6]. Most of these heifers exhibited some degree of associative learning over a period of up to eight test sessions, but the learning rate was highly inconsistent between individuals. Furthermore, similar to previous studies using manual collars [7], the behavioural responses to the collar stimuli were greatly variable across animals. Some individuals showed appropriate behaviours, including stopping and turning away from the stimuli, whereas other individuals showed undesirable responses, such as running forward [6]. Across time, these heifers also appeared to learn the location of the virtual fence, demonstrated by a strong unwillingness to continue approaching the feed reward (i.e., an unwillingness to move much beyond the paddock entry gate), and thus the trials had to be ceased [6]. If cattle are learning the location of a virtual fence, they may not enter a new area when a fence is moved. However, if they are attentive to the audio cue and have learned its association with the electrical stimulus, they should continue forward into a new zone when they approach an old virtual barrier and do not receive any signals. This latter behaviour is critical to the success of virtual fencing technology, but to date has not been assessed in a paddock setting.

The use of some types of virtual fencing systems have elicited welfare concerns, and may not be practical for countries that currently ban the use of electric shock collars for dogs (e.g., Germany, Switzerland, and Wales; [2]). Electric shocks in principle may adversely affect an individual’s welfare, particularly if they are not delivered based on animal behaviour, thus impeding learning by the animal. There are also concerns that collars could lead to ulceration, the equipment might malfunction, or the devices may be inappropriately used [2]. The limited welfare research to date has shown similar short-term stress responses (measured via plasma cortisol, β-endorphin concentrations, heart rate, and behaviours) between steers held in a crush and subjected to either mild unpredictable electric shocks or head bail restraint [8]. The effective design of virtual fencing technology that delivers cues based on both the animal’s location and their behavioural response to cues will appropriately train the animals to associate a non-aversive audio cue (behavioural observations of an animal’s first response to the audio tone indicates its lack of aversiveness [5]) with an aversive electrical stimulus. This associative learning enables the animal, over time, to avoid virtual boundaries based on the non-aversive audio cue alone, thus enabling an ethical implementation of virtual fencing systems [5].

Behavioural patterns and time budgets can also be used as indicators of welfare, and thus these data from longer-term studies (spanning across weeks or more) using automated collar prototype designs are needed. Scan sampling observations of pastured beef cattle have shown typical time budgets to comprise walking, grazing, standing ruminating/resting, and lying resting/ruminating for over 95% of their time [9]. Similarly, as reviewed across multiple cattle studies in varying settings, generally 90–95% of time is spent grazing, ruminating, and resting [10]. Changes from “normal” patterns of behavioural time budgets can indicate welfare issues. For example, different intensive housing conditions can alter the time engaged in specific behaviours between individuals [11]. Reduced lying times in indoor-housed dairy cattle have been reported to be associated with less comfortable lying surfaces [12], where reduced lying times can result in chronic stress [13].

There are limited data available on the application of automated virtual fencing systems longer-term (over several weeks and greater) and the responses of animals to the cues when present within a group. The on-farm application of this technology, and one of its practical benefits, would typically see fences being shifted to aid in pasture management and enable optimal feed efficiency. Thus, the current study was carried out to assess how heifers within a group would respond to a virtual fence line that was periodically shifted to exclude animals from different portions of an experimental paddock, including quantifying any changes in behavioural activity. It was predicted that cattle would avoid a virtual line by responding to the audio cue, but may also exhibit some location-learning. Thus, cattle were expected to move into new zones when they approached the old virtual line and did not receive signals, but only if they did not actively avoid previous virtual fence locations. It was also predicted that behavioural patterns and time budgets would not differ between the different virtual fence periods.

## 2. Materials and Methods

### 2.1. Ethical Statement

All experiments were approved by the CSIRO FD McMaster Laboratory Chiswick Animal Ethics Committee (AEC16/28) prior to the start of experimentation.

### 2.2. The Virtual Fencing Collar

For this experiment, cattle were fitted with re-purposed collar straps (from MooMonitors^®^, Causeway, County Kerry, Ireland) that included approximately 1.5 kg of hanging counterweights (three counterweights were included with variation between individual weights) and the automated experimental prototype virtual fencing collar device (eShepherd^TM^, Agersens, Melbourne, VIC, Australia; device weight: 0.80 kg and 19 cm L × 10 cm W × 5 cm H), positioned on the left side of each animal’s neck. Using GPS technology, the collar device monitors the animal’s movement and provides a real-time measure of the animal’s position, heading, and speed. A virtual fence boundary (separating inclusion versus exclusion zones), specified using GPS coordinates, is transmitted to the collar using a radio frequency link. If movement was above a specific velocity (unable to be stated due to intellectual property rights), i.e., the animal was trotting/running, the animal was deemed unresponsive to stimuli and thus stimuli were not applied. As the animal reaches the virtual fence boundary (directly on the virtual line), the collar device emits a 2.5 s audio cue (single continuous tone at 785 Hz ± 15 Hz, 58 DB). If the animal stands still or turns away, no electrical stimulus is applied. Continuous movement through the virtual fence line elicits an electrical stimulus immediately following the audio cue. For this experiment, an electrical stimulus level of 800 V electrical pulses delivered in less than 1 s was used (precise details are unable to be specified due to intellectual property rights). This sequence of an audio cue followed by the electrical stimulus is repeated if the animal walks through the fence line and continues into the “exclusion zone” (the collar has a temporary shut-down feature if multiple stimuli are applied). No stimuli are applied if the animal turns around to walk back out of the exclusion zone. Based on testing by the experimenters, animals generally have to be within a few feet of each other to be able to hear another collar’s audio tone. The wind and other surrounding noise will muffle the tone even further and reduce the chance that the cattle will hear each other’s devices. Further details on the collar device algorithm are available in the patent description [3,4]. The date, time, and GPS location are logged by the collar device multiple times each minute (precise number varies). All emitted stimuli details are also logged for later download from the device.

### 2.3. Animals and Experimental Protocol

**Figure 1:**
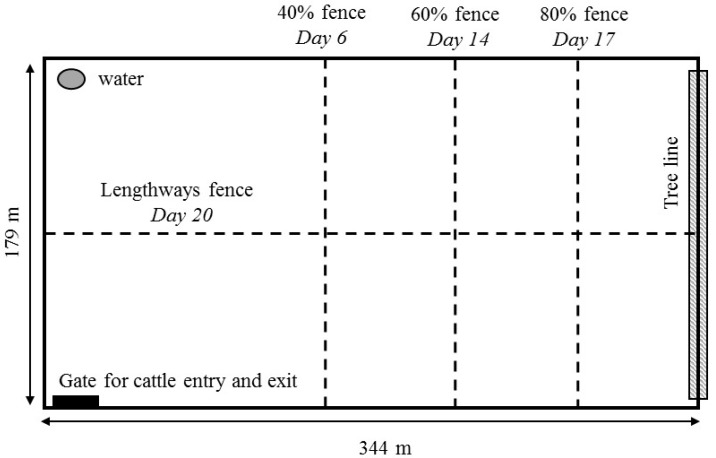
The test paddock for the virtual fencing trial showing the position of the water trough, the tree line overhanging the upper physical fence boundary, and the gate for cattle entry into and exit from the paddock. The dashed lines represent the virtual fence boundaries that were sequentially implemented in the paddock over the trial duration.

On Day 1 of the experimental trial (March 2017), 11 approximately 19 to 20 month-old (September/October 2015 drop) Angus heifers were each fitted with the automated experimental prototype collar. These cattle had recently been tested in a feed attractant trial using the virtual fencing collars [6], and were thus habituated to the collars. The animals were not naïve to the audio and electrical stimuli, but their learning rate in the previous study had been highly variable. The animals refused to continue approaching the virtual fence line before all individuals had clearly demonstrated associative learning [6]. An IceQube^®^ 1.001 (IceRobotics Ltd., Roslin, Midlothian, Scotland, UK) was fitted to each animal’s back left leg to monitor standing, lying, and steps (recorded in 15-min intervals) across the trial duration. All cattle were placed into a rectangular-shaped, 6.15 hectare experimental grassed paddock located in Armidale, NSW. A water trough was present at the bottom of the paddock, and there was a line of trees at the opposite end (Figure 1).

The cattle were given five days to acclimate to the test paddock, with access to 100% of the paddock area. The collars were set to record the GPS position of the cows, but no virtual fence line was set. On Day 6, a virtual line was set across the width of the paddock to restrict the cows to 40% of the paddock area (40% inclusion zone, Figure 1), and supplementary hay was provided at the bottom of the paddock in case of pasture shortage (no formal measures of pasture quantity or quality were made during the trial). Even though animals had some prior exposure to the collar stimuli [6], there was an initial learning period with the fence line and animals crossed over into the exclusion zone. After approximately 48 h, all cattle were mostly restricted within the 40% inclusion zone. However, by Day 11, 5 of the 11 cattle were regularly crossing back into the exclusion zone as their experimental prototype collars malfunctioned (this lead to a split in the herd; the cattle with functional collars correctly stayed within the inclusion zone). These five cattle were removed from the trial on Day 13, and henceforth only six cattle were used. On Day 14, the fence line was moved to restrict animals to 60% of the paddock area, on Day 17 the fence line was moved to restrict animals to 80% of the paddock area, and on Day 20 the fence line was moved to run lengthways down the centre of the paddock (a potentially more complex fencing change), thus restricting the animals to one side of the full paddock length (Figure 1). The trial concluded on Day 22.

Across the trial duration, the batteries on the experimental prototype collars had to be recharged approximately every third day, which involved a removal of the collar. When the battery levels were low, all collars were turned off, and the animals were brought into the cattle race located approximately 70 m adjacent to the test paddock. All animals were checked for neck lesions from the collar device during battery changes and weighed using walk-over scales placed within the crush. The cattle were always herded out and returned through the bottom of the test paddock so that they never walked through the locations of the virtual boundaries, and the collars were turned back on as the animals re-entered the paddock (Figure 1).

Battery charging initially took approximately 24 h each time. Following the removal of the cows with malfunctioning collars (Day 13), spare batteries were used, thus battery changing was reduced to approximately two hours henceforth. Animals were returned to the test paddock if battery charging occurred during the baseline period when no fence was turned on. Once the virtual fences were applied, all cattle were kept in either a holding paddock (63.6 m L × 35.2 m W) adjacent to the cattle yards if the battery charging occurred overnight, or kept in the cattle yards for the shorter changing times. Prior to moving the fence line, it was ensured that the cattle were present in the paddock and had tested the current fence that day (i.e., had approached the fence and received some collar stimuli). This protocol minimised the potential impact of being removed and held in a different location during battery changing on the animal’s learning of the fence. All times of battery charging were excluded from all datasets. Thus, in total, animals were recorded for 115 h with “no fence”, for 137.15 h with the 40% inclusion zone, for 69.15 h with the 60% inclusion zone, for 68.15 h with the 80% inclusion zone, and for 54.75 h with the lengthways inclusion zone. Day 1 of the baseline (no fence) period was excluded from all datasets, as the animals were acclimating to both the paddock and the IceQubes^®^, thus reducing the total data collection time to 91 h in the “no fence” period. However, there were only 69 h of GPS collar data in the “no fence” period compared to 91 h of IceQube^®^ data, as the animals were in the test paddock wearing the IceQubes^®^ on one occasion when the collar batteries were charging.

### 2.4. Data and Statistical Analyses

Data were only included from those six cattle that remained within the experiment for its entire duration. However, one collar stopped logging data during the 80% fence location period and two collars during the lengthways fence location period. These collars were still delivering the correct signals to the animals (personnel regularly checking the animals both in person and with the online system observed no indication that the collars were not delivering the correct signals), but were not recording data and thus could not be included in the analyses. The collar log data were compiled to provide the percentage of time each animal spent in the inclusion zone for each different fence location. The total number of audio and electrical stimuli delivered by the collars was also summed separately for each different inclusion zone period for each animal, and the proportion of audio-only stimuli were calculated. These data are presented in the Results, but due to the low sample size and uneven sample numbers, no statistical analyses were conducted. The total number of crossings over the fence into the exclusion zone were also tallied for each approximate day of each fence location for individual animals. These count data were square-root transformed and compared between inclusion zone periods using a general linear model with repeated measures.

The 15-min IceQube^®^ readings (time spent lying, time spent standing, number of steps, and number of lying bouts) for all six individual animals were summed every two hours across the five different inclusion zone periods. The count data for steps and lying bouts were square-root transformed. All data were analysed using general linear mixed models with repeated measures, including the fixed effect of “inclusion zone” and individual animal as a random effect. All behavioural activity data were averaged across all animals and daily patterns for each inclusion zone period presented in the Results.

All analyses were conducted in JMP^®^ 13.0.0 (SAS Institute, Cary, NC, USA) with α set at 0.05. The raw values are presented in the Results, as there was virtually no difference between the raw and back-transformed data.

## 3. Results

### 3.1. Responses to Virtual Fences

**Figure 2:**
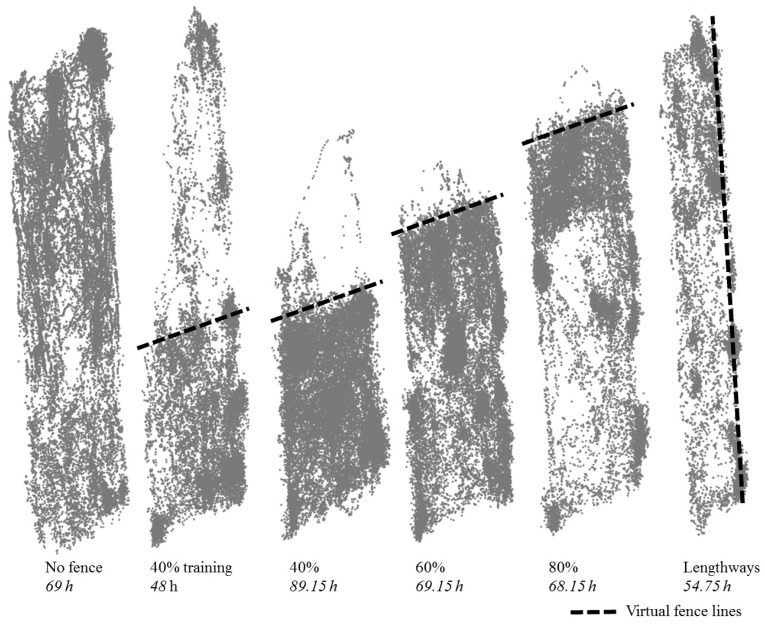
The global positioning system (GPS) positional data from all cattle (*n* = 6) in the test paddock with the different virtual fence lines (dashed lines) set sequentially across a period of 22 days (total time within each fence period indicated in h). An approximately 48-h period within the 40% inclusion zone was separated out to display the learning period. Each point indicates a location a cow was present with some expected GPS error ± approximately 5 m. Due to collar malfunction, data were only recorded from *n* = 5 cows during the 80% fence period and from *n* = 4 cows during the lengthways period; collars were functional and kept the animals within the inclusion zones but did not log data.

No lesions or rubbing from the collars were observed across the trial duration. The cattle increased in body weight across the trial duration (Day 1: mean ± standard error (SEM) body weight 496.67 ± 8.25 kg, Day 20 body weight 517.17 ± 9.47 kg). The cattle accessed all the test paddock area during the time period when no virtual fences were present, with a preference for sleeping by the tree line at night time (Figure 2). There was an approximately 48 h initial learning period within the 40% inclusion zone, and thus overall, the cows spent an average of 88.6% (±SEM 0.02%) of their time within the prescribed area (Figure 2). This increased to an average of 96.70% (±SEM 0.01%) during the 60% inclusion zone period, an average of 98.05% (±SEM 0.03%) during the 80% inclusion zone period, and an average of 98.78% (±SEM 0.01%) during the lengthways inclusion zone period.

**Figure 3:**
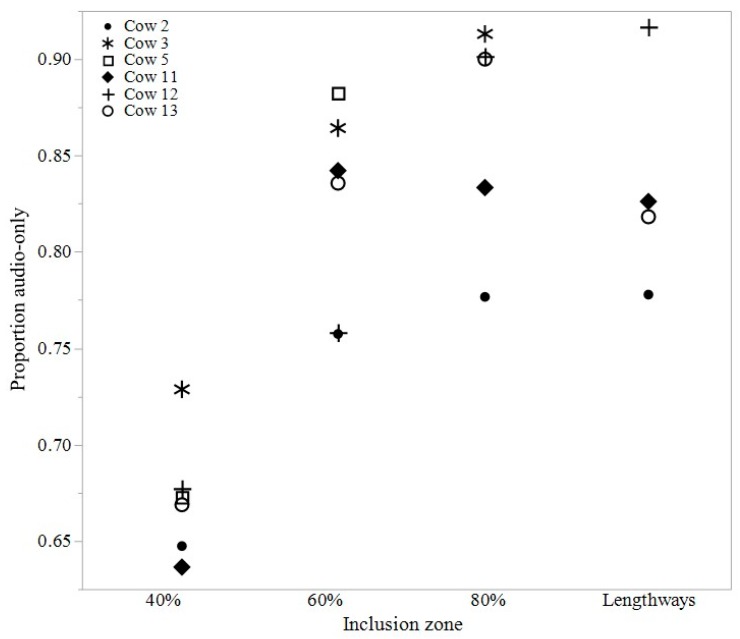
The proportion of audio-only stimuli for individual cattle within each of the four sequential inclusion zones located in a single test paddock. The 40% inclusion zone included the initial period of animal learning. Data from six cattle were recorded during the 40% and 60% inclusion zones, but due to collar malfunction, data were only recorded from *n* = 5 cows during the 80% fence period and from *n* = 4 cows during the lengthways period; collars were functional and kept the animals within the inclusion zones but did not log data.

There was no difference in the average number of times the animals crossed into the exclusion zone between the different fence location periods (*p* = 0.87), with a mean (±SEM) of 7.76 ± 0.51 crossings daily. It was an average of 4 h 15 min until the first cow interacted with the new fence line across the 60%, 80%, and lengthways inclusion zones. The lowest proportion of audio-only cues were emitted during the 40% inclusion zone period (mean ± SEM 0.67 ± 0.01; 60% inclusion zone: 0.82 ± 0.02; 80% inclusion zone: 0.86 ± 0.03; lengthways inclusion zone: 0.83 ± 0.03, Figure 3). There was variation between individual cows in the proportion of audio-only cues received (Figure 3).

### 3.2. Behavioural Activity

**Figure 4:**
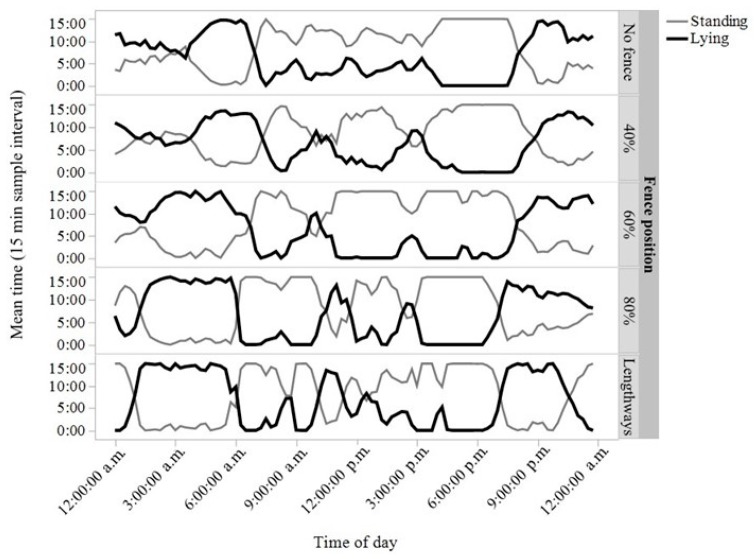
Mean time within 15-min sample intervals that cattle (*n* = 6 heifers) were standing or lying across the day for each of the different positions of the virtual fence (no fence, 40%, 60%, 80%, and lengthways).

**Figure 5:**
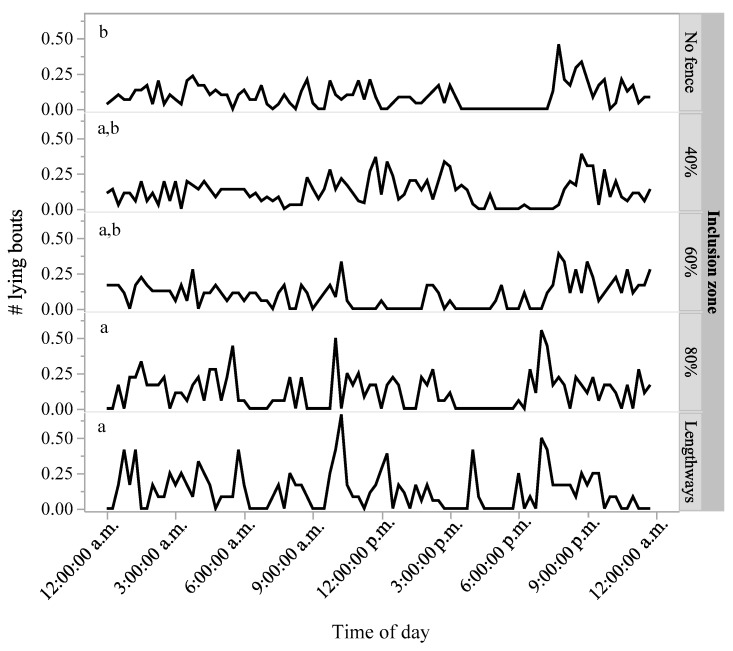
Mean number of lying bouts within 15-min sample intervals throughout the day, averaged across six heifers for each of the different virtual fence inclusion zones (no fence, 40%, 60%, 80%, and lengthways). ^a,b^ Superscript letters indicate significant differences in the number of lying bouts between inclusion zones (*p* < 0.01).

There were no differences in time spent standing (*p* = 0.93), number of steps (*p* = 0.42), or time spent lying (*p* = 0.93) between the different fence location periods (Table 1). The exhibited behavioural patterns of standing and lying were visually similar across the day for the different inclusion zones (Figure 4). There was, however, an effect of fence location on the number of lying bouts (F_(4,1237)_ = 2.55, *p* = 0.04), with more lying bouts during the 80% and lengthways inclusion zone periods compared with the time period where no fence was set (Table 1, Figure 5). The lengthways inclusion zone period showed a 26% increase in number of lying bouts relative to the “no fence” period, but this was not statistically different from the prior varying inclusion zones, indicating incremental changes across time (Table 1, Figure 5).

## 4. Discussion

This trial, using automated experimental prototype virtual fencing collars on Angus heifers in an experimental setting, showed that animals were successfully restricted within specific paddock inclusion zones using GPS-based virtual boundaries. Across a period of approximately 48 h, as predicted, the cattle learned to appropriately respond to the audio cues alone, minimising the number of electrical stimuli they received. Within approximately 4 h, animals accessed new inclusion zones when fence lines had been moved, thus showing that the animals were learning to respond to the audio cues as an indication of the virtual boundaries rather than showing any predicted learning of the boundary location. Overall, as predicted, there were few effects of the virtual fencing system on the animals’ standing, lying, and movement activity.

The practical reality and appeal of virtual fencing technology is that fence lines can be shifted whenever desired. Prior to this study, it was unclear how cattle would respond to moving lines. Location-based learning may lead animals to remain within a specific inclusion zone even after the virtual line has been shifted. In the previous feed attractant trial with the same cattle, over a period of eight individual tests, animals were no longer willing to approach the feed resource, and thus no longer interacted with the fence line [6]. This may have resulted from location-learning of the virtual fence, or a negative association with the hay. Similar results have been found in previous feed attractant trials with manual collars that emitted electrical stimuli only (no audio cues), where cattle over time appeared to associate the feed trough with the electrical stimulus and were no longer willing to approach it [14], or where bulls learned to associate cows with electrical stimuli and avoided them [15]. In the current experiment, on average, within 4.25 h, cattle were first interacting with the new fence line. The cattle were also able to readily adapt to the lengthways (confer widthways) virtual line, which was implemented as a more complex fencing change. These behavioural responses, in combination with a similar number of fence crossings within each inclusion zone, indicate that the animals were regularly testing the fence line and not avoiding a learned location.

Baseline GPS tracking showed a preference for spending time at the top of the paddock, and the motivation to access this area may have also encouraged this continued interaction with the virtual line. Specifically, the animals did not appear to be self-restricted to limited areas of the paddock in a fearful response to a non-visible aversive stimulus. This is in contrast to what has been found in smaller experimental settings, where heifers were trained to avoid specific areas via either electric collars (virtual fences but with no audio cues-manually controlled), or visual electric fences. Following the removal of the physical and virtual barriers, the cattle trained with the virtual fence showed a higher avoidance of the previously learned aversive area [16]. However, experiments in different paddock environments could assess how a preference for an area within the exclusion zone impacts interactions with virtual fence lines. Reduced daily testing of the fence line may result if animals’ preferences for certain locations remain within the inclusion zones.

The relatively rapid movement into new inclusion zones indicated that the animals were attentive to the cues of the virtual fence and were modifying their movement behaviour based on collar signals. This associative learning was anticipated based on the cattle showing the ability to associatively pair audio and electrical stimuli in both a previous experiment using manual collars [5] and in the recent feed attractant experiment using automated collars with the same individuals as the current study [6] (albeit at highly variable individual rates). However, one of the group of six individuals had learned to consistently run forward rather than retreat when tested individually in the previous experiment [6]. Thus, potentially, this individual could have continued to exhibit a learned undesirable response, or their undesirable behaviour could have influenced others within the group, resulting in no animals learning to stay within the inclusion zone. This animal (cow # 5) surprisingly, did not show the lowest proportion of audio-only cues in the current trial (Figure 3). Some recent evidence suggests that cattle in a group at pasture do behave independently of each other [17], and there was a clear split in herd movement in the current trial when five collars malfunctioned. However, the high proportion of time spent within the inclusion zones by all cattle does suggest a positive influence of social facilitation during learning, or a desire to remain with the group that minimised the differences between animals. Audio cues on the collars were set at a volume typically only audible to the individual animal, and thus some degree of independent learning was still exhibited, as all animals responded to the audio cue alone across time. The larger paddock area in the current study (6.15 ha confer approximately 0.25 ha [6]) may have made it easier for the cattle to learn that the desired response was to turn away from the stimulus rather than run forward to escape it, thus facilitating this individual learning. Further study could assess the learning rates of naïve groups of cattle and the potential influences of group hierarchies [18], including whether all individuals need to experience the collar stimuli to be excluded from specific areas.

In the behavioural activity measures taken, few impacts of the virtual boundaries were found. Visually similar behavioural patterns were displayed throughout the day within the different inclusion zone periods, with animals spending approximately half of their time standing, similar to what has been observed in beef cattle at pasture ([12]: half of behavioural scans were standing/grazing), although grazing behaviour was not specifically assessed by the activity monitors in this study. Cattle did show an increase in lying bouts when the fence line moved to include 80% of the paddock or a lengthways portion of the paddock compared to their baseline behavioural patterns, but these changes were incremental across the different inclusion zone periods. Total lying time did not change, and thus the changes in lying bouts only, may indicate that the cattle were slightly unsettled while adapting to the changes in their environment. During the period of no fence, the animals showed a preference for accessing the tree line at the top right end of the paddock to rest under (Figure 2). It is possible that the 80% fence was close enough to the tree line that the cattle were frustrated they could not access it; similarly, the lengthways fence provided access to the trees, but not their preferred corner. A physical electric fence restricting animals to different areas may have elicited similar responses, a potential control condition to be included in future experiments. Furthermore, the original group of cattle was reduced during the 60% inclusion zone, and potential changes in social structure may have led to unrest in the group. The differences that were found may have also just been due to sampling day and general variation across time, irrespective of paddock inclusion zones [19], as there was otherwise no control group for comparison. We might have actually expected a greater behavioural change when the cows had access to different-sized areas of the paddock across time, but the animals appeared to adjust to different paddock zones and maintained similar behavioural patterns. Further study needs to control for effects of days to draw conclusions on the impacts of virtual boundaries on behavioural welfare. Future research should also assess physiological welfare impacts of the collar devices in a paddock setting. These may include measurements of acute stress-induced hyperthermia [20] as cattle are learning the collar stimuli, and further internal body temperature measures when cattle learn to respond to the audio cue alone or heart rate variability as a measure of chronic stress [21].

## 5. Conclusions

Overall, this study in an experimental paddock showed that virtual fencing technology applied via automated collar devices could restrict a single, small group of cattle within specific inclusion zones in a specific paddock setting. In some commercial situations, particularly extensive beef systems, animal inclusion zones may surpass thousands of hectares, fence lines may remain in fixed locations for weeks, and herd numbers may exceed thousands of individuals. Accordingly, these findings should not be generalised to all circumstances, and further study is required using larger groups in different environments. Additionally, future research should assess the effectiveness of virtual fences dependent on the resources animals are being excluded from and their motivation to access them, such as higher pasture quality being present in the exclusion zone. The animals in this study did not appear to learn to avoid the location of the virtual fences, but responded to the cues of the collar with rapid movement into new inclusion zones after the virtual fence line was shifted. Few effects of the virtual fencing system were found on overall activity and resting behaviour, but physiological welfare measures need to be made to understand any negative impacts of the technology.

## Figures and Tables

**Table 1:** Mean activity (±standard error) within 2 h periods of six cattle with different virtual fence inclusion zones * †.

Behaviour	No Fence	40%	60%	80%	Lengthways
Standing time (%)	54.4 ± 2.08	56.5 ± 1.83	54.92 ± 2.5	54.0 ± 2.58	55.0 ± 3.0
# steps	270.8 ± 14.9	276.0 ± 13.0	305.3 ± 17.9	282.7 ± 18.4	340.2 ± 21.6
Lying time (%)	45.6 ± 2.08	43.5 ± 1.83	45.08 ± 2.5	46.0 ± 2.58	45.0 ± 3.0
# lying bouts	0.75 ± 0.05 ^b^	0.90 ± 0.04 ^a,b^	0.81 ± 0.06 ^a,b^	0.93 ± 0.06 ^a^	0.97 ± 0.07 ^a^

* ^a,b^ Different superscript letters for # lying bouts indicate significant differences at *p* < 0.05; † data were summed within 2 h periods across a total of 91 h with no fence, 137.15 h with the 40% inclusion zone, 69.15 h with the 60% inclusion zone, 68.15 h with the 80% inclusion zone, and 54.75 h with the lengthways inclusion zone. Time percentages are of discrete samples taken at 15-min intervals.

## References

[1] Andersen D.M., Estell R.E., Holechek J.L., Ivey S., Smith G.B. (2014). Virtual herding for flexible livestock management—A review. Rangel. J..

[2] Umstatter C. (2011). The evolution of virtual fences: A review. Comput. Electron. Agric..

[3] Lee C. (2006). An apparatus and method for the virtual fencing of an animal. International Patent.

[4] Lee C., Reed M.T., Wark T., Crossman C., Valencia P. (2010). Control device, and method, for controlling the location of an animal. International Patent.

[5] Lee C., Henshall J.M., Wark T.J., Crossman C.C., Reed M.T., Brewer H.G., O-Grady J., Fisher A.D. (2009). Associative learning by cattle to enable effective and ethical virtual fences. Appl. Anim. Behav. Sci..

[6] Campbell D.L.M., Lea J.M., Haynes S.H., Farrer W.J., Leigh-Lancaster C.J., Lee C. (2017). Virtual fencing of cattle using an automated collar in a feed attractant trial. Appl. Anim. Behav. Sci..

[7] Bishop-Hurley G.J., Swain D.L., Anderson D.M., Sikka P., Crossman C., Corke P. (2007). Virtual fencing applications: Implementing and testing an automated cattle control system. Comput. Electron. Agric..

[8] Lee C., Fisher A.D., Reed M.T., Henshall J.M. (2008). The effect of low energy electric shock on cortisol, β-endorphin, heart rate and behaviour of cattle. Appl. Anim. Behav. Sci..

[9] Kilgour R.J., Uetake K., Ishiwata T., Melville G.J. (2012). The behaviour of beef cattle at pasture. Appl. Anim. Behav. Sci..

[10] Kilgour R.J. (2012). In pursuit of “normal”: A review of the behaviour of cattle at pasture. Appl. Anim. Behav. Sci..

[11] Elmore M.R., Elischer M.F., Claeys M.C., Pajor E.A. (2015). The effects of different flooring types on the behavior, health, and welfare of finishing beef steers. J. Anim. Sci..

[12] Schütz K.E., Cox N.R. (2014). Effects of short-term repeated exposure to different flooring surfaces on the behavior and physiology of dairy cattle. J. Dairy Sci..

[13] Fisher A.D., Verkerk G.A., Morrow C.J., Matthews L.R. (2002). The effects of feed restriction and lying deprivation on pituitary-adrenal axis regulation in lactating cows. Livest. Prod. Sci..

[14] Lee C., Prayaga K., Reed M., Henshall J. (2007). Methods of training cattle to avoid a location using electrical cues. Appl. Anim. Behav. Sci..

[15] Lee C., Prayaga K.C., Fisher A.D., Henshall J.M. (2008). Behavioral aspects of electronic bull separation and mate allocation in multiple-sire mating paddocks. J. Anim. Sci..

[16] Markus S.B., Bailey D.W., Jensen D. (2014). Comparison of electric fence and a simulated fenceless control system on cattle movements. Livest. Sci..

[17] Stephenson M.B., Bailey D.W. (2017). Do movement patterns of GPS-tracked cattle on extensive rangelands suggest independence among individuals?. Agriculture.

[18] Šárová R., Špinka M., Panamá J.L.A., Šimeček P. (2010). Graded leadership by dominant animals in a herd of female beef cattle on pasture. Anim. Behav..

[19] Robért B.D., White B.J., Renter D.G., Larson R.L. (2011). Determination of lying behavior patterns in healthy beef cattle by use of wireless accelerometers. Am. J. Vet. Res..

[20] Sanger M.E., Doyle R.E., Hinch G.N., Lee C. (2011). Sheep exhibit a positive judgement bias and stress-induced hyperthermia following shearing. Appl. Anim. Behav. Sci..

[21] Kovács L., Kézér F.L., Jurkovich V., Kulcsár-Huszenicza M., Tőzsér J. (2015). Heart rate variability as an indicator of chronic stress caused by lameness in dairy cows. PLoS ONE.

